# Beyond latent and active tuberculosis: a scoping review of conceptual frameworks

**DOI:** 10.1016/j.eclinm.2023.102332

**Published:** 2023-11-17

**Authors:** Syed M.A. Zaidi, Anna K. Coussens, James A. Seddon, Tamara Kredo, Digby Warner, Rein M.G.J. Houben, Hanif Esmail

**Affiliations:** a WHO Centre for Tuberculosis Research and Innovation, Institute for Global Health, University College London, UK; b MRC Clinical Trials Unit at University College London, UK; c Department of Public Health, National University of Medical Sciences, Pakistan; d Division of Infectious Diseases and Immune Defence, Walter and Eliza Hall Institute of Medical Research, Australia; e Department of Medical Biology, University of Melbourne, Australia; f Centre for Infectious Diseases Research in Africa, Institute of Infectious Disease and Molecular Medicine, University of Cape Town, South Africa; g Department of Infectious Disease, Imperial College London, UK; h Desmond Tutu TB Centre, Department of Paediatrics and Child Health, Stellenbosch University, South Africa; i Health Systems Research Unit, South African Medical Research Council, Cape Town, South Africa; j Molecular Mycobacteriology Research Unit and Division of Medical Microbiology, Department of Pathology, University of Cape Town, South Africa; k TB Modelling Group, TB Centre, London School of Hygiene and Tropical Medicine, UK

**Keywords:** Tuberculosis, Conceptual framework, Spectrum, Latent tuberculosis, Incipient tuberculosis, Subclinical tuberculosis

## Abstract

There is growing recognition that tuberculosis (TB) infection and disease exists as a spectrum of states beyond the current binary classification of latent and active TB. Our aim was to systematically map and synthesize published conceptual frameworks for TB states. We searched MEDLINE, Embase and EMcare for review articles from 1946 to September 2023. We included 40 articles that explicitly described greater than two states for TB. We identified that terminology, definitions and diagnostic criteria for additional TB states within these articles were inconsistent. Eight broad conceptual themes were identified that were used to categorize TB states: State 0: *Mycobacterium tuberculosis* (*Mtb*) elimination with innate immune response (n = 25/40, 63%); State I: *Mtb* elimination by acquired immune response (n = 31/40, 78%); State II: *Mtb* infection not eliminated but controlled (n = 37/40, 93%); State III: *Mtb* infection not controlled (n = 24/40, 60%); State IV: bacteriologically positive without symptoms (n = 26/40, 65%); State V: signs or symptoms associated with TB (n = 39/40, 98%); State VI: severe or disseminated TB disease (n = 11/40, 28%); and State VII: previous history of TB (n = 5/40, 13%). Consensus on a non-binary framework that includes additional TB states is required to standardize scientific communication and to inform advancements in research, clinical and public health practice.

**Research in context**

**Evidence before this study**

In recent years, there has been growing recognition that the binary classification of tuberculosis (TB) into “latent” and “active” does not accurately reflect the complex pathophysiology of the disease process and that it may also be inadequate for informing research and programmatic advances for global TB elimination. While a number of articles have proposed multiple states of infection and disease, no previous study has mapped and synthesized evidence from published literature to inform an overarching and inclusive staging framework. We conducted a comprehensive search on MEDLINE, Embase and EMCare databases for systematic or narrative review articles or commentaries with terms related to TB and “states”, “stages,” “paradigm” “framework” or “spectrum” without date restrictions.

**Added value of this study**

To our knowledge, this is the first study to systematically review conceptual frameworks, terminologies and diagnostic criteria for TB states beyond the latent and active paradigm. We identified that there is substantial variation in the number of TB states described in the literature, as well as in the concepts used to categorize them. Terms used for describing TB states and their diagnostic criteria were also inconsistently applied.

**Implications of all the available evidence**

This review led to and supported the development of the First International Symposium on New Concepts in Early TB Disease (ICE-TB) that aimed to reach expert consensus on a framework for multiple TB states and their terminologies. It is anticipated that this framework will help standardize how TB disease is reported in literature, inform clinical and public health management as well as guide research for TB elimination.

## Introduction

An estimated 10.6 million individuals fell ill with tuberculosis (TB) in 2021 and the disease caused 1.6 million deaths globally.^1^ TB elimination priorities, diagnostic approaches and treatments for the past several decades have been based on a binary understanding of the disease, described as having either latent infection or active (clinical) disease.^2^ While this simplicity has facilitated the development of programmatic management of TB since it was introduced over three decades ago, it has several limitations. Recent understanding of TB has revealed a more complex pathophysiology with a spectrum of disease states, ranging from the time of exposure to *Mycobacterium tuberculosis* (*Mtb*) bacilli to symptomatic, “active” disease.^3^ Individuals within this spectrum likely differ in their prognostic and treatment outcomes, however, their clinical management continues to be based on a binary understanding of the disease.^4^ Individuals with early and unrecognized TB states may also be an important source of community transmission and are therefore critical from an epidemiological perspective.^5^ From a programmatic viewpoint, individuals with early disease can be potentially misdiagnosed or missed by the health system. This is becoming increasingly relevant as active case-finding interventions are being scaled-up across high-burden countries.^6^

Numerous articles have been published recently motivating that TB should be considered as a spectrum of disease states. However, authors often take different perspectives (e.g. immunological, microbiological, public health) and it is currently unclear which states have been commonly described in literature, what terms have been used to describe them, how should they be defined, as well as what diagnostic tests can be used to identify them. There is a need to map TB states beyond latent and active disease described in the literature to help guide researchers, clinicians and policymakers. Therefore, the aim of this scoping review was to collate the terminology, conceptual and diagnostic definitions utilized to describe the spectrum of TB states beyond the latent and active paradigm.

## Methods

### Review objectives

The scoping review was conducted based on a protocol that was registered at the Open Science Framework (OSF).^7^ The review consisted of the following research questions: 1*) what novel conceptual states have been described for TB beyond latent and active disease and how can they be categorized; 2) what nomenclature has been used to describe novel TB states; 3) what are the diagnostic definitions for novel TB states; 4) which conceptual states of TB have been described as potentially infectious and as representing TB disease; and 5) how have the concepts and terms used for TB states have changed over time.* Due to the lack of working definitions and absence of conceptual clarity on TB disease states, a scoping review methodology was considered appropriate. The protocol was developed in accordance with the recommendations provided by the Joanna Briggs Methods Manual for Scoping Reviews.^8^ The scoping review objectives were developed with input from all authors. The inclusion criteria and charting methods were developed by one author (SMAZ) and reviewed by another author (HE) to improve the validity. The study protocol was developed by two authors (SMAZ and HE) and reviewed by the remaining authors. The Preferred Reporting Items for Systematic reviews and Meta-Analyses Extension for Scoping Reviews (PRISMA-ScR) was utilized to guide the reporting.^9^

### Search strategy and data sources

To address research questions 1–4, we conducted a systematic search on MEDLINE, Embase and EMcare databases on Ovid for terms related to TB states, stages or spectrum. The search was period was from 1946 to September 2023. The complete list of terms utilized in the scoping review are described in Supplementary Table S1. A filter for “Review Articles” was applied in Ovid. This is because the aim of this scoping review was to synthesize evidence for descriptions of TB as a multi-state disease rather than to systematically identify all articles with a non-binary staging in literature. A restriction to review articles allowed for inclusion of diverse perspectives across disciplines while also ensuring feasibility of the review. The search strategy was developed with input from a specialist librarian at the UCL Great Ormond Street Institute of Child Health Library. In addition to the database search for published review articles, to address research question 5, we separately charted the history of classification of TB used by the National TB Association (NTA) of the United States of America, from its foundation in 1904 to the current day. Transactions of the annual meetings of the NTA and the published Diagnostic Standards (in there various formats) from 1917 to 2016 were obtained.^10^ The findings from this historical review are summarized in Box 1.

Box 1 **TB Disease states: historical perspective.**

The articles that form part of this scoping review are all recent which in part reflects the search strategy, selection of databases and inclusion criteria. However, discussions and debates on the classification of TB have been ongoing since well before Koch's discovery of *Mycobacterium tuberculosis*. Historical literature and the Diagnostic Standards and Classification of Tuberculosis of the (American) National Tuberculosis Association (published periodically since 1917) highlight an evolving classification of TB driven by changing priorities for TB control, available diagnostics and treatments.^10^

From the early nineteenth century, authors referred to and discussed the merits of staging systems for TB with terminology such as *phthisis incipiens* (incipient TB), *phthisis confirmata* (confirmed TB) and *phthisis desperata* (desperate/severe TB) in use.^11^^,^^12^ By the early twentieth century there were numerous staging systems reflecting different perspectives, for instance based on pathology, diagnosis, extent, presentation or disease course.^12^, ^13^, ^14^ It was recognized that there was a tension between complex classification systems capturing multiple dimensions of the disease accurately vs. simplistic approaches, such as the 3-stage Turban-Gerhardt classification, based on disease extent that was could be more easily applied and hence widely implemented but ultimately proved unsatisfactory.^13^^,^^14^ Another simplified approach consisted of describing tuberculous lesions as “open” or “closed.” In open TB, the lumen of the draining bronchus was considered to be patent, allowing for potentially greater release of bacilli and sputum expectoration.^15^ Closed TB lesions were described as having an occluded bronchus, leading to atelectasis and scar formation, thereby rendering the individual non-infectious. This classification also proved inadequate, particularly with regards to infectiousness of an individual.^16^

While TB continued to be largely managed in sanatoria, microbiological, radiological and other diagnostic tools to assess the state of disease were becoming increasingly available and the need for a standardized approach to disease classification was recognized to aid statistical comparisons and clinical communication. In particular, it was considered important to have a consistent approach to classifying the condition of the patient at entry into the sanatoria (reflecting prognosis) and the condition during follow-up and at discharge (reflecting the degree of cure/healing and likelihood of relapse). Soon after inception of the National Tuberculosis Association in 1904, a *Committee on Clinical Nomenclature* was formed.^17^^,^^18^ Discussions, surveys and consensus-building for over a decade led to the development of the 1st Diagnostic Standards and Classification of Tuberculosis in 1917.^19^ Here the classification of pulmonary TB in adults consisted of 3 dimensions each clearly defined; (i) the extent of pulmonary lesions initially categorized as incipient, moderately advanced and far advanced (ii) symptoms categorized slight/none, moderate (iii) clinical/treatment status—initially suspicious case, definite case, arrested/apparently arrested case. Over subsequent iterations details were added, clinical status became more nuanced, bacteriology was increasingly emphasized and there were changes to terminology. However, the broad classification approach remained similar with a focus on stratification by disease extent and clinical status.^18^

The availability of antibiotics from the 1950's for both curative and preventive therapy resulted in a radical change in the approach to managing TB and eventually led to a shift in disease classification.^20^ Prognostic considerations and clinical status became less relevant as treatment remained the same for all individuals and a prolonged follow-up for relapse was not required. The 13th edition of the Diagnostic Standards in 1974 were a major departure from previous classifications highlighting 5 states; *0: No tuberculosis exposure, not infected, I: Tuberculosis exposure, not infected, II: Tuberculosis infection, without disease, III: Tuberculosis infection, with disease, IV: Tuberculosis suspect*.^20^ Central to this classification was diagnosis of infection requiring evidence of immune sensitization without evidence of disease (symptoms, radiological or microbiological) and of disease through microbiological confirmation. Finally in 1999, the term *latent* was prefaced to *tuberculosis infection, no disease* to distinguish from *clinically active tuberculosis*, leading to the binary paradigm in use to the present day.^21^

### Inclusion criteria

Eligible articles explicitly described TB as a multi-state disease, i.e., provided description of states beyond the existing two-state latent and active disease paradigm. Included articles consisted of reviews pertaining to a range of topics including TB pathogenesis, symptomatology, immunology and bacteriology, diagnostics, vaccinology and transmission. Only articles published in the English language were eligible for inclusion. Additional articles not identified through the search were considered for inclusion based on the recommendations of the authors if they provided descriptions of TB as a multi-state disease. These articles were not limited to reviews. Articles describing specific diagnostic approaches or biomarkers without describing distinct conceptual states were excluded. Articles limited to descriptions of TB pathology or bacteriology without describing TB conceptual states within individuals were excluded. Articles that focused specifically on differing pathophysiology or clinical presentation of childhood or extra-pulmonary TB only, without describing a staging framework for TB were excluded. However, articles describing these disease types within the wider context of TB as a multi-state disease were included.

### Screening, data abstraction and charting

Outputs from the Ovid searches from the three databases were uploaded on Covidence and duplicates were removed.^22^ Two authors (SMAZ and HE) independently reviewed abstracts for inclusion. Conflicts were resolved through discussions based on the inclusion and exclusion criteria. Full-text articles were assessed for eligibility by one author (SMAZ) and reviewed by a second (HE). Conflicts on inclusion of articles after full-text reviews were resolved through review by a third author (RMGJH).

The data-charting table was developed on MS Excel. Summary characteristics of the included articles, textual descriptions of TB states, concepts and terminologies were abstracted. Conceptual descriptions of TB states described as part of diagrams or within texts for each article were tabulated. Due to the variation in frequency, descriptions and terminologies utilized for disease states between articles, textual descriptions with common concepts were thematically derived and coded. These conceptual themes were labelled as distinct “*states*” and were subsequently categorized numerically. Extended descriptions or minor variations within themes were separately coded in order to capture all described states and were categorized alphanumerically. Frequency of each state and their variations among included articles were calculated. Two authors (SMAZ and HE) developed the coding and all authors reviewed the development of the final descriptions of TB states. Data-abstraction and coding was conducted by two authors (SMAZ and HE) and conflicts on the coding were resolved by mutual discussion. Disagreements on coding for TB states were resolved by a third author (RMGJH). One author (HE) reviewed proceedings from the NTA and abstracted historical timelines for the usage of various terminologies for describing TB disease states.

To record diagnostic criteria, outcomes of diagnostic tests and symptomatology described for each state were extracted from the articles. These were categorized as positive, positive/negative (i.e., test could be either positive or negative), negative, or not described. Frequency statistics describing diagnostic tests and symptomatology as positive or positive/negative for each state were calculated. Frequency of articles describing each state as potentially infectious and as representing TB disease were also calculated.

### Role of funding source

The funders had no role in the design, data analysis or writing of this manuscript.

## Results

### Literature search

**Fig. 1:**
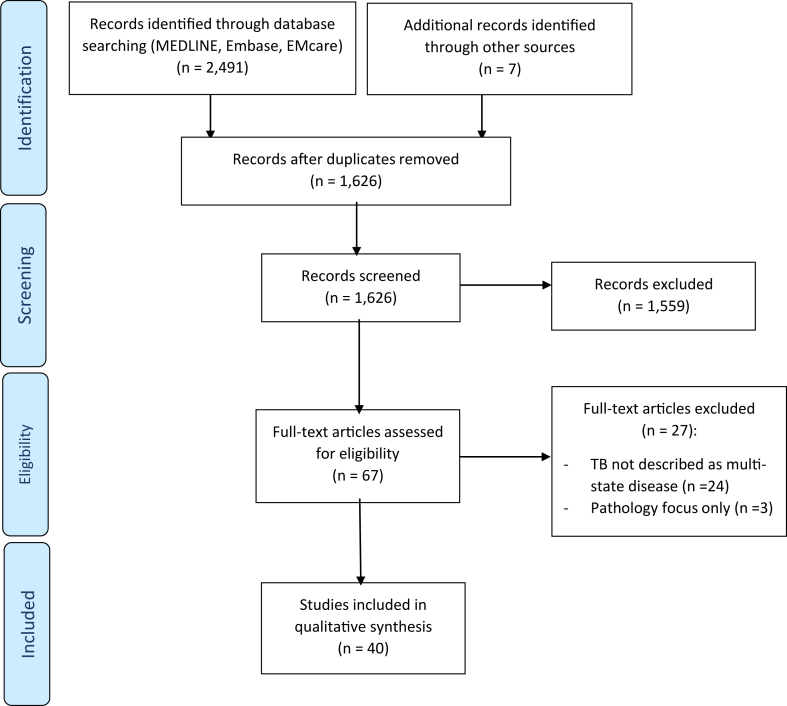
Selection of sources of evidence.

A total of 2491 articles were identified through the database search (Fig. 1). A further seven articles were identified through recommendations of the contributing authors, including three perspective articles and one related to epidemiological modeling. After removing duplicates, 1626 article abstracts were screened, from which 67 full-text articles were assessed for eligibility. A total of 40 articles were included in the final qualitative synthesis. Most excluded articles (89%, n = 24/27) did not describe TB as a multi-state disease. Articles focused on a singular aspect of the disease, such as pathology without describing states that would be of clinical or epidemiological relevance, were also excluded (11%, n = 3/27). Nearly all of the included articles had conceptual diagrams with descriptions outlining disease states and their transitions.

### Characteristics of the included articles

Most articles consisted of narrative reviews (83%, n = 33/40) (Table 1). Three articles (8%) were perspectives, two were systematic reviews and one a mathematical model. Most articles were focused on novel understandings of TB pathogenesis (43%, n = 17/40). The remaining articles described novel diagnostic approaches for TB (28%, n = 11/40), advances in vaccine development (8%, n = 3/40), epidemiological or transmission implications of early TB states (15%, n = 6/40), radiological findings of early TB (3% n = 1/40) and treatment strategies (3%, n = 1/40). Most articles (93%, n = 37/40) were written by primary authors based in high-income countries.

**Table 1:** Characteristics of the articles included in the scoping review.

Reference	Year	Title	Article aims & objectives	Type of article
Barry 3rd et al.^3^	2009	The spectrum of latent tuberculosis: rethinking the biology and intervention strategies	Review of biology of latent TB, host responses and drug development	Review article
Young et al.^23^	2009	Eliminating latent tuberculosis	Review of heterogeneity in TB physiology and host immune response and impact on therapeutic and diagnostic approaches	Review article
Chao and Rubin^24^	2010	Letting sleeping dos lie: does dormancy play a role in tuberculosis?	Description of in vitro models of TB dormancy and resuscitation during infection.	Review article
Achkar and Jenny-Avital^25^	2011	Incipient and Subclinical Tuberculosis: Defining Early Disease States in the Context of Host Immune Response	Review of early TB disease states and their impact on biomarker, diagnostics and drug discovery	Review article
Gideon and Flynn^26^	2011	Latent tuberculosis: what the host “sees”?	Review of human and non-human primate models of TB that have led to new concepts of latent TB	Review article
Kunnath-Velayudhan et al.^27^	2011	Immunodiagnosis of Tuberculosis: a Dynamic View of Biomarker Discovery	Review of clinical spectrum of Mtb infection, evolution of granulomatous lesions and granuloma structural changes	Review article
Lawn et al.^28^	2011	Changing Concepts of “Latent Tuberculosis Infection” in Patients Living with HIV Infection	Review of spectrum of TB bacteriology and immunology and implications for HIV associated TB	Review article
Sridhar et al.^29^	2011	Redefining latent tuberculosis	Review of latent TB biology, immunology and diagnostics	Review article
Esmail et al.^30^	2012	Understanding latent tuberculosis: the key to improved diagnostic and novel treatment strategies	Review of current diagnosis and treatment of latent Mtb infection and developments in understanding the biology of latency	Review article
Joel D. Ernst^31^	2012	The immunological life cycle of tuberculosis	Descriptive framework for investigating immunity to Mtb to guide vaccine discovery and development.	Review article
Delogu et al.^32^	2013	The biology of mycobacterium tuberculosis infection	Review of advances in TB biology and pathogenesis and evolving paradigm of latent Mtb infection	Review article
Dowdy et al.^33^	2013	Is Passive Diagnosis Enough? The Impact of Subclinical Disease on Diagnostic Strategies for Tuberculosis	Estimating impact of population level case finding strategies for subclinical TB	Mathematical Epidemic Model
Delogu and Goletti^34^	2014	The Spectrum of Tuberculosis Infection: New Perspectives in the Era of Biologics	Review of TB disease spectrum	Review article
Esmail et al.^35^	2014	The ongoing challenge of latent tuberculosis	Review of current understanding of latent TB and highlight novel diagnostic, drug regimens and vaccines development approaches	Review article
Nunes-Alves et al.^36^	2014	In search of a new paradigm for protective immunity to TB	Review of advances in immune control of Mtb and implications for vaccine design and evaluation	Review article
Salgame et al.^37^	2015	Latent tuberculosis infection—Revisiting and revising concepts	Host, clinical and epidemiologic features of latent Mtb infection and diagnostic development	Review article
Pai and Behr^38^	2016	Latent *Mycobacterium tuberculosis* Infection and Interferon-Gamma Release Assays	Review of tests for latent TB diagnosis	Review article
Pai et al.^39^	2016	Tuberculosis	Review of the global TB epidemic	Review article
Petruccioli et al.^40^	2016	Correlates of tuberculosis risk: predictive biomarkers for progression to active tuberculosis	Review of biomarkers for progression to active TB	Review article
Cadena et al.^41^	2017	Heterogeneity in tuberculosis	Review of TB biology and heterogeneity observed in the human disease	Review article
Drain et al.^42^	2018	Incipient and Subclinical Tuberculosis: a Clinical Review of Early Stages and Progression of Infection	Review of current understanding of TB pathogenesis, immunology, clinical epidemiology, diagnosis, treatment, and prevention	Review article
Kik et al.^43^	2018	An evaluation framework for new tests that predict progression from tuberculosis infection to clinical disease	Descriptive framework for generating evidence to inform the development of policy guidance for tests for incipient TB	Review article
Lin et al.^44^	2018	The End of the Binary Era: Revisiting the Spectrum of Tuberculosis	Review of data supporting the spectrum of Mtb infection in humans and nonhuman primates	Review article
Sousaa and Saraiva^45^	2018	Paradigm changing evidence that alter tuberculosis perception and detection: Focus on latency	Review of new understandings of TB latency to inform research and elimination	Review article
Houben et al.^46^	2019	Spotting the old foe—revisiting the case definition for TB	Novel disease case definitions for TB	Perspective
Lewinsohn and Lewinsohn^47^	2019	New Concepts in Tuberculosis Host Defense	Review of TB immunology and novel host-directed therapies	Review article
McHenry et al.^48^	2020	Genetics and evolution of tuberculosis pathogenesis: New perspectives and approaches	Descriptive framework on possible transitions from pathogen exposure to TB disease and associated genetics studies	Review article
Boom et al.^49^	2021	The knowns and unknowns of latent *Mycobacterium tuberculosis* infection	Review of immune responses to Mtb in humans and nonhuman primates, new concepts and major knowledge gaps in understanding of latent Mtb infection	Review article
Kendall et al.^50^	2021	The Epidemiological Importance of Subclinical Tuberculosis A Critical Reappraisal	Conceptual framework for subclinical TB and transmission	Perspective
Migliori et al.^51^	2021	The definition of tuberculosis infection based on the spectrum of tuberculosis disease	Review of TB as a disease spectrum	Review article
Alebouyeh et al.^52^	2022	Feasibility of novel approaches to detect viable *Mycobacterium tuberculosis* within the spectrum of the tuberculosis disease	Review of feasibility of new approaches to detect TB bacilli across the full spectrum disease	Review article
Chandra et al.^53^	2022	Immune evasion and provocation by *Mycobacterium tuberculosis*	Review of TB biology, immunity and development of host-directed therapies, biomarkers and vaccines.	Review article
Esmail et al.^2^	2022	Mind the gap. Managing tuberculosis across the disease spectrum.	Review of TB as a spectrum of disease states, new diagnostic approaches and clinical trials	Review article
Houben et al.^5^	2022	Tuberculosis prevalence: beyond the tip of the iceberg	Novel stages and disease thresholds for TB	Perspective
Scriba et al.^54^	2022	Challenges in TB research	Challenges in developing new diagnostics and vaccines for TB control	Perspective
Yoon et al.^55^	2022	CT and 18F-FDG PET abnormalities in contacts with recent tuberculosis infections but negative chest X-ray	Systematic review of CT and PET-CT imaging of asymptomatic TB contacts with normal X-rays	Systematic review
Sossen et al.^4^	2023	The Natural History of Untreated Pulmonary Tuberculosis in Adults: A Systematic Review and Meta-Analysis	Quantify progression and regression across the spectrum of TB disease of individuals with untreated TB during the pre-chemotherapy era	Systematic Review
Agbota et al.^56^	2023	Management of Tuberculosis Infection: Current Situation, Recent Developments and Operational Challenges	Review of recent developments in management of LTBI and operational challenges	Review
Escalante et al.^57^	2023	New diagnostics for the spectrum of asymptomatic TB: from infection to subclinical disease	Review of developments and challenges in diagnostics for LTBI and the spectrum of asymptomatic TB	Review
Kim et al.^58^	2023	Host Blood Transcriptional Signatures as Candidate Biomarkers for Predicting Progression to Active Tuberculosis	Review of progress and challenges in predictive blood transcriptional signatures	Review

All the articles were published from 2009 onwards, with 16 (40%) being published during or after 2020. This reflects the relative novel conceptualization of TB as a multi-state disease within contemporary literature. However, as described in Box 1, such conceptual states were recognized and described in the pre-antibiotic era.

### Conceptual definitions of TB states

A total of eight distinct conceptual themes were identified that were categorized as TB states (Table 2). We categorized the first state as **State 0** and this was described in 25 (63%) articles. The conceptual theme for this state was elimination of *Mtb* with innate immunity due to absence of detectable acquired immune memory to *Mtb* infection. Uninfected individuals were also categorized as State 0. The most common terms for this state were ‘*innate immune response”, “innate immune elimination”* and *“uninfected.”* **State I** described in 31 (78%) articles was conceptually similar to State 0 in that the infection was eliminated. However, the elimination was described as involving an acquired immune response. This host response was frequently described as detectable and these individuals were categorized as distinct from those who were uninfected. The most used terms for this state were “*acquired immune,” “adaptive immune response*” and “*latent tuberculosis infection (LTBI)*.” Individuals in **State II** described in 37 (93%) articles were categorized as those in whom *Mtb* has not been eliminated, however, the infection was described to be controlled by the immune system. This state was most commonly described as “*quiescent infection*”, “*latent tuberculosis infection*” and “*incipient TB*”. **State III** described in 24 (60%) articles was categorized as individuals in whom there was a failure to control the infection or a breakdown of immune control. The most commonly used terms for this state were *“incipient”, “incipient TB infection”,* and *“incipient TB disease.”* **State IV** described in 26 (65%) articles categorized individuals that were bacteriologically positive for TB but had no symptoms, or had symptoms that were not recognized or were not seeking care for symptoms. The state was most frequently described as *“subclinical”* or *“subclinical disease”*. In **State V,** individuals had TB signs and symptoms and were actively seeking care, in the presence or absence of microbiological confirmation. This state was described in 39 (98%) of articles. This state was described as *“active TB disease”, “clinical TB”* or as *“disease.”* **State VI** described in 11 (28%) articles was differentiated from the previous state through terms describing increased disease severity such as cavitary disease, disseminated or extensive disease. The most frequently utilized term for this state was “*Cavitary/Disseminated*”. **State VII** grouped all individuals with a previous history of TB including those previously diagnosed, treated, or cured. This state was least frequently described, featuring in only 5 articles (13%). Articles varied in the use of conceptual themes to describe the TB spectrum (Table 3).

**Table 2:** TB states conceptual definitions and terminology.

State	Conceptual theme	Terminology used to describe state	Articles describing state (n)	%
State 0	Infection has been eliminated with innate immune response	**Uninfected, Innate immune Response, Innate immune elimination,** No Disease (TB Negative), Eliminated TB infection, Infection controlled/eliminated/Stable, Uninfected individual, No *M. tuberculosis* infection, Cleared, Latent Clearance, Uninfected, Initial infection, TB infection, Innate Immune Cell Recruitment, Colonization and Early Clearance, Infection, Resistance to Infection, Stage 1, Elimination of infection (self-resolving infection), Infection eliminated (with innate immune)	25	63%
State I	Infection has been eliminated by acquired immune response	**Acquired Immune, Adaptive Immune Response, LTBI**, No Disease (Latent TB), Acquired Immune Response, Acquired Immune Elimination—memory, LTBI—Asymptomatic, Eliminated TB infection, Acquired Immune, Infection controlled/Eliminated/Stable, Non-progression or Self-cure from Subclinical Stage, Primary or secondary *M. tuberculosis* infection, LTBI—Reverter, Latent Clearance, Reduced Reactivation Risk, Adaptive Immune Phase, Transient Infection, Adaptive Immune Cell Activation, Resister, Clearance of Infection, Mtb infection, Latent TB infection, AsymptomaticLTBI—Incident	31	78%
State II	Infection is not eliminated but is controlled by the immune system	**Quiescent Infection, Latent Mtb infection**, **LTBI, Incipient TB,** Latent Infection, No Disease (Latent TB), Quiescent, Unstable LTBI, Latent Non-infectious tuberculosis, Mtb infection, Immunological equilibrium (latency), Latent Subclinical or Low Grade TB, Primary Infection, Latent Infection Dormant/Persistent, Traditional LTBI, Incipient TB, Caseating granuloma, Persistent TB, Containment of infection (Established Latency)	37	93%
State III	Infection is not controlled by the immune system	**Incipient, Incipient TB infection, Incipient TB disease,** No Disease (Latent TB), Active Infection, Subclinical TB Disease, Asymptomatic, Incipient disease, Unstable LTBI Incipient, Infectious Tuberculosis, Active Severe TB, Resuscitation, Reactivation TB, Percolating Infection, Progressor/Incipient TB, Stage 2, Failure to Contain Infection (subclinical active TB), Culture-Negative TB	24	60%
State IV	Individual is bacteriologically positive but has no symptoms or clinical features	**Subclinical, Subclinical TB Disease**, **Asymptomatic**, Bacteriologically Positive, Subclinical Active Phase, Subclinical No Symptoms, Additional Infectious Tuberculosis, Subclinical TB with no Signs/Symptoms, Subclinical Disease, Subclinical/Incipient, Active Infection, Incipient TB, Asymptomatic Pre-clinical, Stage 3, Failure to contain infection (Primary Active TB), Subclinical Infection, Quiescent	26	65%
State V	Individual has signs or symptoms associated with TB	**Active TB Disease, Clinical TB, Disease (Active TB), Disease,** Minimal, Active TB DiseaseSymptomatic Clinical Bacteriologically Negative, Pre-diagnostic TB Disease, Clinical TB, Subclinical Symptoms not Recognized, Clinical Disease (Symptoms) Infectious Period, Active Clinical TB Disease, Chronic TB, Reactivation/Secondary TB, Reactivation, TB Disease, Transmission, Active TB, Smear Negative, Active TB Caseating Granuloma, At Risk for TB, Primary Active Disease, Active TB, Symptomatic—Clinical, Stage 4, Breakdown of Immune Control (Active TB), Definite TB	39	98%
State VI	High severity of disease due to clinical features, radiology or bacteriology	**Cavitary/Disseminated**, **Active TB DiseaseSymptomatic Clinical Smear Positive/Extensive Disease**, **Dissemination disease**, Active, Severe, Symptomatic Culture PositiveSmear Positive,	11	28%
State VII	Previous History of TB	**Asymptomatic Past TB**, **No Disease (Past TB), Treated/Recovered,** Treated, Old Disease, Relapse, Reinfection	5	13%

Footnote: Three most frequently used terms for each state are highlighted in bold.

Abbreviated terms have been included as described in the article text, tables or diagrams.

Tuberculosis (TB), *Mycobacterium tuberculosis* (Mtb), Latent Tuberculosis Infection (LTBI).

**Table 3:** TB states described by articles included in the review.

Reference	State 0	State I	State II	State III	State IV	State V	State VI	State VII
Barry 3rd et al. (2009)	X	X	X	X		X		
Young et al. (2009)	X	X	X	X		X		
Chao and Rubin (2010)		X	X	X		X		
Achkar and Jenny-Avital (2011)			X	X		X	X	X
Gideon and Flynn (2011)	X	X	X		X	X	X	
Kunnath-Velayudhan et al. (2011)			X		X	X	X	X
Lawn et al. (2011)	X	X	X		X	X		
Sridhar et al. (2011)	X	X	X	X		X		
Esmail et al. (2012)	X	X	X		X	X	X	
Joel D. Ernst (2012)	X		X	X			X	
Delogu et al. (2013)	X		X			X		
Dowdy et al. (2013)			X		X	X		X
Delogu and Goletti (2014)	X	X	X			X		
Esmail et al. (2014)		X	X	X	X	X		
Nunes-Alves et al. (2014)		X	X			X		
Salgame et al. (2015)	X	X	X	X	X	X	X	
Pai and Behr (2016)	X	X	X		X	X	X	
Pai et al. (2016)	X	X	X		X	X		
Petruccioli et al. (2016)	X	X	X	X	X	X		
Cadena et al. (2017)		X	X	X		X		
Drain et al. (2018)	X	X	X	X	X	X		
Kik et al. (2018)		X	X	X	X	X		
Lin et al. (2018)	X	X			X	X	X	
Sousaa and Saraiva (2018)	X	X	X	X		X		X
Houben et al. (2019)		X	X	X	X	X		
Lewinsohn and Lewinsohn (2019)	X	X	X		X	X		
McHenry et al. (2020)	X	X	X			X		
Boom et al. (2021)	X	X	X	X		X	X	
Kendall et al. (2021)	X	X	X	X	X	X		X
Migliori et al. (2021)	X	X	X		X	X		
Alebouyeh et al. (2022)		X		X	X	X		
Chandra et al. (2022)	X	X	X			X	X	
Esmail et al. (2022)			X	X	X	X	X	
Houben et al. (2022)				X	X	X		
Scriba et al. (2022)	X		X		X	X		
Yoon et al. (2022)		X	X	X	X	X		
Sossen et al. (2023)			X	X	X	X		
Agbota et al. (2023)	X	X	X	X	X	X		
Escalante et al. (2023)	X	X	X	X	X	X		
Kim et al. (2023)		X	X	X	X	X		

Within the eight conceptual states, we identified an additional 27 unique variations (Table 4). These were minor differences within the conceptual themes that we had used to categorize states. For States 0-I, conceptual variations included an acquired immune response that was detectable vs. no longer detectable and elimination of infection through administration of preventative treatment. Conceptual variations for States II-IV differed in diagnostic results such as detection of granulomas on imaging. State V had the largest number of variations with articles differing in their descriptions for detection on imaging, bacteriological positivity and care-seeking behavior.

**Table 4:** Variations within TB states identified from articles included in scoping review.

State	Concept	n	(%)
**State 0**	**Infection has been eliminated by innate immune response**	**25**	**63%**
State 0A	Innate resisters (individuals naturally resistant to acquiring infection)	8	20%
**State I**	**Infection has been eliminated by acquired immune response**	**31**	**78%**
State IA	Infection eliminated through specific immune mechanism that is detectable	17	43%
State IAa	Infection eliminated through specific immune mechanism that is no longer detectable (reversion)	12	30%
State IAb	Infection eliminated through specific immune mechanism >2 years	1	3%
State IB	Infection eliminated through TPT	3	8%
State IBb	Infection eliminated through TPT (INH)	5	13%
State IBb	Infection eliminated through TPT (RIF)	5	13%
**State II**	**Infection is not eliminated but is controlled by the immune system**	**37**	**93%**
State IIA	Infection is not eliminated, controlled in granuloma (not detectable, replicating bacteria)	13	33%
State IIB	Infection is not eliminated, controlled in granuloma (detectable)	3	8%
State IIC	Infection is not eliminated, controlled in granuloma (not detectable, non-replicating bacteria)	15	38%
**State III**	**Infection is not controlled by the immune system**	**24**	**60%**
State IIIA	Infection is not eliminated, not controlled in granuloma (detectable)	10	25%
**State IV**	**Individual is bacteriologically positive but has no symptoms or clinical features**	**26**	**65%**
State IVA	Detectable bacteria but no symptoms (additional sampling)	2	5%
State IVB	Detectable bacteria but no symptoms (Bac + ve, CXR -ve)	14	35%
State IVBa	Detectable bacteria but no symptoms (Bac + ve, CXR + ve)	14	35%
**State V**	**Individual has signs or symptoms associated with TB**	**39**	**98%**
State VA	Symptomatic or Clinical Disease (Bac -ve)	6	15%
State VAa	Symptomatic or Clinical Disease (Bac -ve, CXR -ve)	1	3%
State VAb	Symptomatic or Clinical Disease (Bac -ve, CXR + ve)	4	10%
State VB	Symptomatic or Clinical Disease (Bac + ve)	25	63%
State VBa	Symptomatic or Clinical Disease (Bac + ve, CXR -ve)	1	3%
State VBb	Symptomatic or Clinical Disease (Bac + ve, CXR + ve)	16	40%
State VBc	Symptomatic or Clinical Disease (Bac + ve, smear -ve)	4	10%
State VC	Symptomatic but not care seeking	6	15%
**State VI**	**High severity of disease due to clinical features, radiology or bacteriology**	**11**	**28%**
State VIA	Disseminated disease	8	20%
State VIB	Cavitary Disease	10	25%
**State VII**	**Previous History of TB**	**5**	**13%**
State VIIA	Repeat Diagnosis after previously cured of TB (recurrent)	2	5%
State VIIB	Cured after TB Diagnosis	5	13%
State VIIC	Death	2	5%

Footnote: TB Preventative Treatment (TPT).

Isoniazid (INH). Rifampicin (RIF). Bacteriologically positive (Bac + ve). Chest X-ray (CXR).

Bacteriologically negative (Bac -ve).Tuberculosis (TB).

### Diagnostic criteria of TB states

**Fig. 2:**
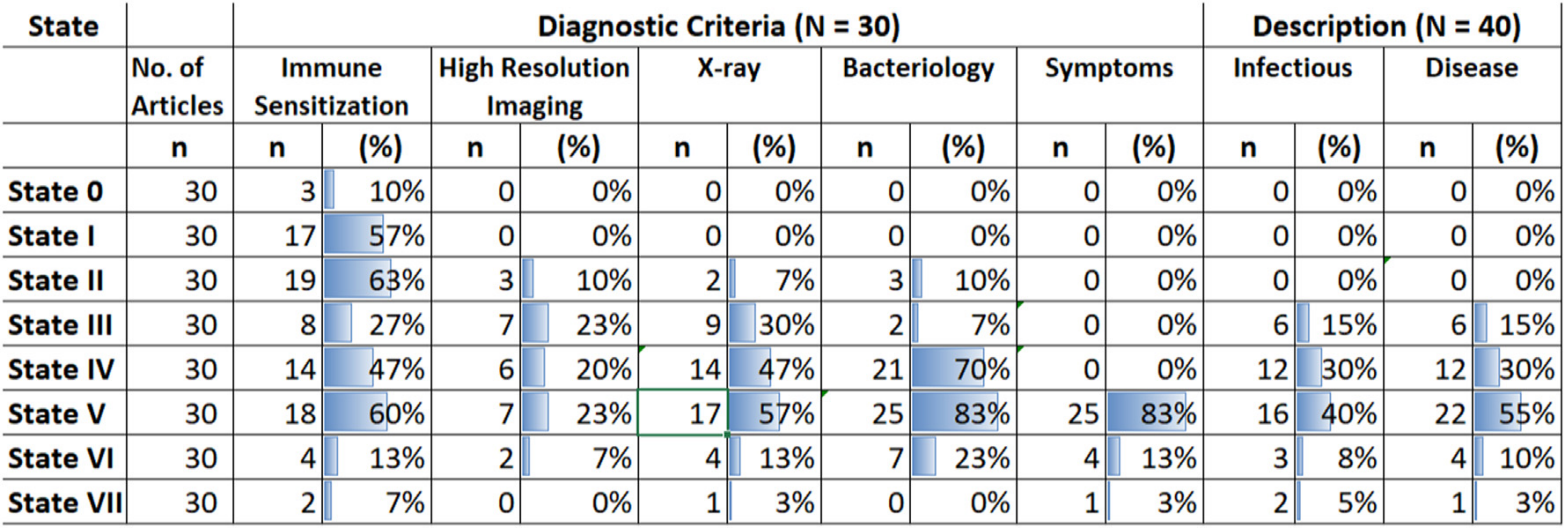
Diagnostic criteria and description of TB states. ∗Number of articles (n) is total articles describing each TB state as positive or positive/negative on diagnostic tests, presence of symptomatology and whether the state was described as infectious or as disease. High resolution imaging included computerized tomography (CT) and positron emission tomography combined with computerized tomography (PET-CT). Total number of articles describing diagnostic criteria was 30 using which the proportions were calculated. Bacteriology includes positive or positive/negative on TB culture, smear or molecular tests. All articles were included in the denominator for proportions for infectious and disease.

Diagnostic criteria for TB states were described on the basis of tuberculin skin testing (TST), interferon-gamma release assay (IGRA), computerized tomography (CT), positron emission tomography combined with computerized tomography (PET-CT), chest X-ray (CXR), sputum culture, smear and Xpert MTB/RIF and symptomatology. Articles varied in the use of diagnostic tests to differentiate states. Due to the variation in the descriptions of diagnostic tests described, we coded these into 5 “diagnostic domains”. These domains were: 1) Immune sensitization (including IGRA, TST); 2) High-resolution imaging (CT and PET-CT); 3) CXR; 4) bacteriology (including all sputum based investigations) and 5) symptomatology. The proportion of articles describing each diagnostic domain as either positive or positive/negative are displayed in Fig. 2. Diagnostic definitions or symptomatology were described in 30 (75% of total) articles either part of diagrams, tables or within texts. Diagnostics were frequently not described for all states and not every diagnostic domain was included in articles. Articles where the outcome of a diagnostic test or symptom screen was not explicitly stated were categorized as missing and not included in the numerator. Articles where some diagnostic information was provided but had missing information for a particular diagnostic for a state have been retained in the denominator.

**State 0** was described as negative on all diagnostic tests with the exception of 3 (n = 3/30; 10%) articles describing positive/negative results on immune sensitivity tests. Individuals in **State I** were most frequently described as having a positive diagnostic result to immune sensitization tests (n = 17/30; 57%) and negative to all other tests. Limited diagnostic differentiation was described for **State II** with articles most frequently describing positive immune sensitivity tests (n = 19/30; 63%), followed by positive CT/PET-CT (n = 3/30; 10%)). **State III** was most frequently described to be positive on CXR (n = 9/30; 30%) and PET-CT/CT (n = 7/30; 23%). **State IV** was frequently described as bacteriologically positive (n = 21/30; 70%) with remaining articles describing this state as subclinical only, without providing explicit diagnostic results. **State V** was most commonly described as having positive symptoms (n = 25/30; 83%) with the remaining articles not explicitly stating whether this state would yield positive symptom screen results. **State VI** was most frequently associated with positive bacteriology (n = 7/30; 23%) and symptoms (n = 4/30; 13%). **State VII** was most frequently associated with positive immune sensitivity (n = 2/30; 7%).

### Infectiousness and disease threshold

To assess the epidemiological significance of each disease state, articles were reviewed for the terms *“infectious”* or *“transmissible.”* To understand at which state articles considered TB disease to begin (disease threshold), the term *“disease”* was reviewed. These terms were included in the terminology, narrative descriptions or as part of conceptual diagrams for disease states. No article attributed the term infectious or disease to describe States 0, I or II. States IV and V were most frequently described as infectious (n = 12/40; 30% and n = 16/40; 40% respectively) and as disease (n = 12/40; 30% and n = 22/40; 55% respectively).

## Discussion

This is the first review to systematically map and synthesize evidence from published literature for additional TB states beyond the prevailing latent and active paradigm. We identified 40 articles relevant to our inclusion criteria, over a third of which were published within the past three years, suggesting increasing interest and recognition of additional states for TB. There are several key themes that emerged from our review.

There was significant variation in the frequency of TB states and in their conceptual definitions, highlighting an overall lack of consensus within literature. Articles varied in their focus and emphasis, for example, TB pathogenesis and bacteriology, immunology, clinical or epidemiological relevance, diagnostic biomarkers and imaging were utilized as different perspectives for the basis of TB states. These perspectives framed the conceptual approach towards TB states and subsequent inclusion or exclusion of particular states. The choice of which states to include may also have been driven by prevailing knowledge gaps and research priorities at the time of the article publication. Earlier articles (2009–2015/16) were broadly more focused on TB immunology and host mechanisms of sterilization of *Mtb* and have therefore described states with innate (the initial, usually non-specific immune response to a pathogen) and adaptive (specific cell-mediated immune response to a pathogen) clearance. More recent articles tended to shift focus towards epidemiological considerations including detection and transmission implications of asymptomatic disease. As a result, states describing subclinical disease were more common. While we aimed to define a parsimonious set of states grouped around common concepts, there were clear variations within these themes. Certain articles chose to further elaborate upon these variations than others and this again reflected the focus of the article and the perspective of the authors.

The review also highlights a lack of consistency in the terminologies used to describe TB states. We observed that not only were different terms utilized to describe the same concept within the natural history of disease but similar terms were also utilized to describe different conceptual states. As examples, bacteriologically positive disease in the absence of symptoms was variously described as *subclinical, asymptomatic, incipient* and *bacteriologically positive*. Similarly, *latent TB* was utilized to describe elimination of infection with an identifiable acquired immune response, containment of infection, as well as breakdown of immune control. Such variation in nomenclature naturally leads to confusion in communication and conduct of scientific research and in public health policy. The understanding of a certain term in literature is subjective to the perspective of the authors and interpretation of the readers and these may not necessarily be the same.

Two-thirds of articles outlined diagnostic criteria. Similar to the conceptual states, articles varied in the choice of diagnostic tests to differentiate between states. The lack of standardized diagnostic criteria to differentiate between TB states may lead to further challenges in describing TB states. We chose to group diagnostic criteria within five domains and these may prove useful in developing diagnostic definitions for proposed conceptual states of TB. Further considerations include achieving consensus on defining the domains themselves. Bacteriology may be defined through culture, molecular tests, smear, as well as type of sampling, such as through sputum, aerosols or through use of induced and invasive methods. Similarly, symptomatology can include those who are truly asymptomatic, those who test negative on a symptom-screen test or those who are symptomatic but do not seek clinical care.

From a public health and individual perspective respectively, the point at which TB is considered infectious to others and the point at which it is considered as causing disease (and hence harm to the individual) is important. It is notable therefore that the majority of articles did not directly address these concept. Infectiousness of TB states was described by less than half of articles. No article had described State 0, I or II as infectious, with this proportion steadily increasing from State III (infection not controlled by immune system) to State IV (bacteriologically positive in the absence of symptoms) and State V (clinically apparent symptomatic TB). It is unclear whether the authors thought the states not described as infectious were truly incapable of leading to secondary infections. Surprisingly, no articles explicitly describe where they believed the disease threshold to begin and this could only be inferred from terminology used in the text and in diagrams. The number of articles using the term disease again increased from State III through to State V.

While it is clear that there is an increasing recognition of the TB spectrum in recent literature, the various states outlined by the included articles are not all new. As our historical review of the NTA proceedings highlights, TB in the past has been considered as having several stages, with staging systems reflecting the public health needs of the time. Several of these early disease states that have been highlighted in the contemporary literature we reviewed were also described in the pre-antibiotic era (Box 1). In the absence of effective treatments, it was recognized that screening, early-identification and prompt isolation of individuals with TB in sanatoria may help control the disease. Development of potent chemotherapy for TB, coupled with increased commitments for primary healthcare, shifted the focus away from mass X-Ray based case finding towards passive approaches towards case-finding and treatment.^59^ This cemented the binary conceptualization of TB, as individuals were either considered to be harboring “latent” infection or presenting to healthcare facilities with clinically apparent, “active” disease. It is possible that the reemergence of early TB disease states in literature over the past decade has coincided with increased investments for active case-finding for TB, such as through community-wide screening and use of mobile X-rays. In addition, improved understanding of TB pathophysiology as well as advances in development of biomarkers and imaging may have stimulated further interest in earlier disease states.

This review led to and supported the development of the First International Symposium on New Concepts in Early TB Disease. The symposium aimed to develop consensus on a framework for TB states and their terminologies. The consensus process involved a broad range of experts and stakeholders ranging from academics, clinicians, policymakers and people with lived experience of TB. A wide representation was necessary so that agreed definitions and terminologies were acceptable from research, clinical, public health and policy perspectives, in addition to being acceptable to individuals with TB. The results of this review not only underscored the need for such a consensus framework but the putative states that emerged from the review helped develop an evidence-base from which such a framework could be developed. The consensus process consisted of a Delphi exercise with two online surveys from invited participants and a series of in-person voting rounds. Participants voted on the acceptability and relevance of the conceptual states described in this review as well as on their preferred terminology for proposed states. Gaps in clinical and diagnostic criteria for disease states that emerged from this review, particularly for States II–V, were also highlighted during the symposium. This helped identify research priorities and the pathway towards clinical and public health applications of a novel TB states framework. It is hoped that the development of a novel disease framework will spur research and innovation to help realign the prevailing paradigm to a more accurate reflection of the disease process. With agreement on conceptualization and shared language, appropriate research questions around diagnostics, treatments and prevention may be developed.

TB has a complex pathophysiology and as we have shown it is possible to describe many states, hence the emergence of the commonly used phrase of “spectrum”. The binary representation of TB is clearly insufficient, however, incorporating any additional states must have a purpose and relevance to public health or to people with TB and should also facilitate the goal of global TB elimination. The review highlights that parsimony and simplicity should remain key guiding principles with clear language, diagnostic definitions and disease thresholds. This will be of critical importance in communication to researchers, care providers, people with TB and to the general public.

Our search strategy was restricted to review articles in English and it is likely that additional TB states or terminology may have been described in other types of articles and languages. However, since the aim of the scoping review was to provide an overview rather than to comprehensively document all described states, this restriction was considered appropriate.

As with other types of thematic analyses, the categorization and coding for TB states is to a degree subjective to the authors’ interpretation and views. This is particularly relevant for states where conceptual diagrams were not provided and categorization had to be inferred from the article text. Our goal was to categorize states around a minimum number of common concepts, not all variations described in the articles could be adequately captured within one of these categories. We attempted to resolve this by categorizing and quantifying variations within these descriptions. Biases in coding were minimized by coding through two reviewers and resolution by a third reviewer in cases of disagreement. All study authors reviewed the final set of states and their diverse expertise and backgrounds helped further minimize bias.

Articles varied in the diagnostic tests and criteria utilized to classify diseases states depending on the nature of the article and background of the authors. This led to a number of articles with missing information on diagnostics, symptomatology, disease description and transmission potential for those disease states. To remove potential for biases, a diagnostic test or criteria was not included in the positive and positive/negative category unless explicitly stated in the article. The ratios of positivity on diagnostic criteria are therefore lower than would be expected had each article described them and should therefore be interpreted with caution and not as precise measurements of the authors’ opinions.

Early states of TB beyond latent and active disease have been increasingly described in literature over the past decade. However, the conceptual definitions and diagnostic criteria for these states and the terminology used to describe them is highly inconsistent. It is anticipated that the proposed novel framework will help standardize how early TB disease is reported in literature, inform clinical and public health management as well as guide research for TB elimination.

### Outstanding questions

Developing diagnostics that are able to differentiate between states will be essential for a novel framework to be meaningful clinically. In particular, tests that can discriminate between States 0, I, II and III identified in our review, i.e., clearance of infection, presence of viable bacilli and containment of infection and breakdown of immune control, have critical clinical and public health significance. Similarly current approaches towards treatment that are based on a binary paradigm will need to be adapted including potentially novel regimens or varying durations of existing regimens. Outcomes of vaccinology studies may require reconsideration with a clearer understanding of which states are being targeted and prevented through administration of a vaccine. While the First International Symposium on New Concepts in Early TB Disease aimed to develop consensus around a novel framework with consistent definitions and terminologies, it is important that this framework is regularly updated with advancements in our understanding of the disease process. This can potentially include states for post-TB lung disease that were not a part of this review.

## Contributors

AKC, HE and RMGJH conceptualized the review and acquired funding. SMAZ and HE conducted the literature search, abstract screening and full text reviews with support from RMGJH. SMAZ and HE conducted data extraction and analysis. All authors contributed to the writing, reviewing and editing of the manuscript. All authors read and approved the final version of the manuscript.

## Data sharing statement

The data used in this scoping review are existing literature and publicly available.

## Declaration of interests

AKC received funding from the Center for Infectious Diseases Research in Africa (CIDRI-Africa), National Institutes of Health and the Bill and Melinda Gates Foundation to support the 1st International Symposium on New Concepts in Early TB. DW has received funding from National Institutes of Health, South African Medical Research Council, Bill & Melinda Gates Foundation and Research Council of Norway. HE is a Data Safety Monitoring Board member for the StatinTB Trial.
