# Supplementary material for: Beyond latent and active tuberculosis: a scoping review of conceptual frameworks
Source: eClinicalMedicine. 2023 Nov 17;66:102332. doi: 10.1016/j.eclinm.2023.102332 (PMC10772263; doi:10.1016/j.eclinm.2023.102332)
Supplement: Supplementary Table S1 [file mmc1.docx]

## Supplementary File

### Supplementary Table 1: Search terms utilized in Ovid in scoping review

| 1 | *Mycobacterium tuberculosis/ or *Latent Tuberculosis/ or *Tuberculosis, Pulmonary/ or *Tuberculosis/ |
| --- | --- |
| 2 | spectrum.mp. |
| 3 | framework.mp. |
| 4 | concept*.mp. |
| 5 | paradigm.mp. |
| 6 | stage.mp. |
| 7 | state.mp. |
| 8 | 2 or 3 or 4 or 5 or 6 or 7 |
| 9 | 1 and 8 |
| 10 | "Review Literature as Topic"/ or "Systematic Review"/ or "Review"/ |
| 11 | 9 and 10 |
